# Effectiveness of an educational intervention in the integral care of patients with diabetes mellitus

**DOI:** 10.12688/f1000research.159626.4

**Published:** 2026-09-21

**Authors:** Evelyn Goicochea-Rios, Nélida Milly Otiniano

**Affiliations:** 1 Essalud Red Asistencial La libertad, La Libertad, Trujillo, 13007, Peru; 2 Universidad Cesar Vallejo, Trujillo, La Libertad, Peru

**Keywords:** Diabetes mellitus Type 2, Health education, Comprehensive approach, self-management; self-care; glycaemic control.

## Abstract

**Background:**

To evaluate the effectiveness of an educational intervention in the integral care of patients with diabetes mellitus.

**Methods:**

A pre-experimental study was carried out with 103 patients with diabetes mellitus treated at Essalud, La Libertad, during the second quarter of 2024. Pre and post educational intervention information was collected about knowledge of the disease, complications, physical activity and diet, adherence to treatment and family support, number of medical and nursing controls per quarter, glycemia values, HbA1c and compliance with treatment after informed consent. The educational intervention included theoretical aspects of diabetes mellitus, self-care, physical activity, nutrition and family support in 2-hour workshops, once a week, for 2 months.

**Results:**

Predominantly female, mean age 63.22 years, higher education, marital status married/cohabiting and main occupation employed/independent. 97.1% had type 2 diabetes mellitus, 38.8% reported disease duration < 5 years, 72.8% were treated with metformin and the main comorbidity was hypertension (58.3%). The educational intervention improved knowledge about diabetes mellitus (p < 0.001), achieved a significant change in eating habits (p = 0.022) and family support (p = 0.043), and homogenized the level of support among families (p = 0.025).

51.5% of the participants were noncompliant with the prescribed treatment. A significant difference (p < 0.01) was observed in the proportion of patients complying with treatment before and after the intervention. 76.8% of patients who initially complied with treatment continued to do so after the intervention. The efficacy of the educational program on glycemic control was not evidenced. Regarding integral control, 83.5 % of the patients received quarterly medical care and 44.7 % received nursing care.

**Conclusion:**

The educational program was effective in improving knowledge about T2D, adherence to healthy eating and significantly improved the level of family support and compliance with treatment.

## Introduction

Diabetes mellitus (DM) is a chronic metabolic disease with heterogeneous etiology and varied clinical presentations. Its microvascular and macrovascular complications affect various organs and systems and the patient’s quality of life due to the costs associated with it. ^1^ It has a high prevalence worldwide ^2^ and increasingly affects age groups under 40 years of age. It is related to being overweight or obesity, sedentary lifestyle and consumption of processed foods. It is also reported that between 50 and 70% of DM cases are not controlled. DM was the sixth leading cause of death in Peru in 2018 ^3^ and in the American continent, it occupied the same place as a cause of death in 2019. ^2^

Older adults with type 2 DM are at greater risk of presenting comorbidities and geriatric syndromes ^4, 5^ and many live alone; factors that complicate the metabolic control of diabetes. ^4^ There is a worldwide consensus that a decrease in the frequency of complications is achieved when the patient takes responsibility for the care of his/her disease and the best strategy to achieve this is education of both the patient and the family. ^6– 8^ For this reason, research is oriented towards the study of programs that through the approach of clinical, educational, behavioral and psychosocial aspects, help to develop positive health behaviors in patients, to meet the treatment objectives, decision making and the guidelines required for optimal self-care of DM. ^7, 9^

Adherence to pharmacological treatment can be influenced by the patient’s beliefs, culture and level of education, but also by the way in which the physician communicates and informs, so it is important to analyze these factors to contribute to metabolic control ^10^: fasting glycemia between 80-130 mg/dL; HbA1c < 7%; blood pressure <130/80 mm Hg; LDL cholesterol <100 mg/dL; HDL cholesterol >50 mg/dL and triglycerides <150 mg/dL. ^11^

Health systems promote comprehensive care for people with chronic disease, which requires the participation of physicians, nurses, nutritionists, social workers, psychologists, as established in the family and community-based model of the Peruvian Ministry of Health. ^12^ Likewise, ESSALUD promotes the standardized and progressive health care model to guarantee the follow-up of people with chronic pathology. This model places the patients as co-responsible for their care, especially in their lifestyles, and as the center of a system for the delivery of comprehensive and interprofessional services over time. ^13^

The integral care of a diabetic patient includes health education with emphasis on behavioral aspects such as daily exercise, healthy eating, glycemic control and medical check-ups. Preventive behaviors include a physical activity plan or a healthy diet, i.e., patients and their families should be aware of carrying out these activities and know how to do them, incorporating the new knowledge into their daily activities. ^14^ Therefore, the educational intervention was considered part of the protocol for caring for patients with diabetes mellitus.

This research is of particular importance, as it attempts to understand the personal, family and socio-cultural context of patients with diabetes mellitus attending a primary care centre, with regard to their level of knowledge about T2D and its complications, their beliefs about the condition, their diet, physical activity, family support in preparing meals and attending medical check-ups, and their level of adherence to treatment.

It is also of practical relevance to standardize educational interventions in workshops adapted to the existing reality in charge of the health team, so the aim of the research was to evaluate the efficacy of an educational intervention in the integral care of the patient with diabetes mellitus through the pre and post intervention analysis of knowledge about DM, dietary habits, type and frequency of physical activity, metabolic control, adherence to treatment and family support.

## Methods

A quantitative, pre-experimental design study with pre-test and post-test was conducted. The population consisted of 580 outpatients with T2D attended per month in family medicine at Hospital I Albrecht - Essalud during the second semester of 2024.

Participants were recruited from patients attending the health center during the study period. Eligibility was defined a priori according to the following criteria:

Inclusion criteria: Adults aged 40–79 years of either sex with a confirmed diagnosis of T2D were considered eligible. Participants were required to have preserved physical and cognitive autonomy, be receiving treatment with oral hypoglycemic agents either as monotherapy or in combination with insulin, provide written informed consent, and attend at least 80% of the scheduled study sessions.

Exclusion criteria: Patients with advanced or disabling chronic complications—defined as blindness, limb amputation, heart failure, or stage 4 chronic kidney disease—were excluded. Additionally, patients who were referred to other healthcare facilities during the study period were not included in the analysis.

The sample size was 150 participants, and it was calculated with the formula for population proportions considering a confidence level of 95%, a test power of 80%. ^15^ Forty-seven patients dropped out: 15 did not attend all the scheduled workshops, 10 because they did not have someone to accompany them to the analysis, 4 because they traveled, 7 because they did not perform the post-intervention analyses, and 7 because they withdrew their informed consent; for this reason, the results of 103 patients who completed the entire program are presented. The Figure 1 illustrates this information.

**Figure 1.**
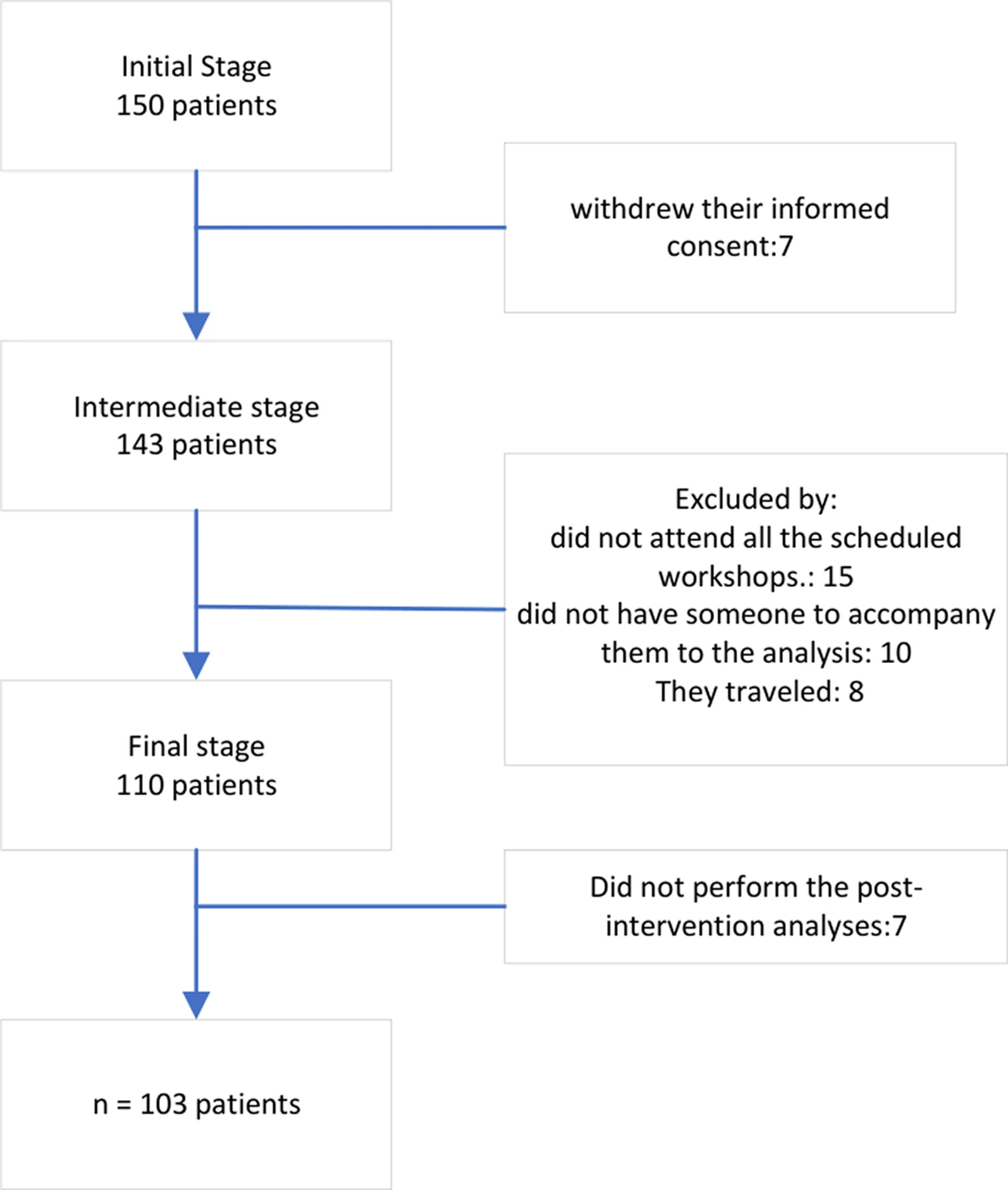
Flowchart diagram of patient recruitment, exclusions, and final sample included in the analysis.

Consecutive non-random sampling was performed, ^16^ since participants were invited to participate in the study on the day they came to the appointment and the sampling frame consisted of the lists of patients with T2D scheduled for outpatient family medicine consultations, in morning and afternoon shifts, six days a week and until the sample was complete.

Pre and post educational intervention, the variables level of knowledge about diabetes, eating habits, glycemic control, adherence to treatment and family support were analyzed.

Regarding comprehensive care, the following variables were analyzed pre and post intervention: fasting glycemia, glycosylated hemoglobin, medical and nursing control attendance, pharmacological treatment compliance report, healthy eating report, physical activity and family support.

### Procedures and techniques

Permission was requested by the institution to carry out the present study, to have access to clinical histories and to evaluate the patients who gave informed consent. The participants were informed of the objectives of the educational program. A form was designed to collect sociodemographic and clinical information on each patient, and it included the tests required to obtain data on the variables under study, which are detailed in the following paragraphs. All the information collected was recorded in an Excel spreadsheet for subsequent analysis.

Before starting the educational program, tests were applied to identify the level of knowledge about T2D (Diabetes Knowledge Questionnaire - DKQ 2417), ^17^ the eating style and physical activity questionnaire, ^18, 19^ adherence to treatment using the Morisky-Green test ^20, 21^ and to identify family/social support (Valadez test ^22^). Pre- and post-intervention fasting glycemia and post-intervention Hba1c were also analyzed. Information collected from medical records regarding adherence to treatment was also recorded.

DKQ2417 consists of 24 questions whose answers comprise the alternatives ‘Yes’, ‘No’ or ‘I don’t know’; one point is obtained for each correct answer, and the final score is the sum of the points. Adequate knowledge: score of more than 13 or more than 55% of correct answers and inadequate knowledge: score of less than 13. ^17^

The eating style and physical activity questionnaire was designed to identify dietary and nutritional risk factors in older adults with T2D. It is interpreted according to whether eating habits are healthy or unhealthy: Healthy habits: Answer yes to questions 2,5,6, unhealthy habits: Answer yes to questions 1,3,4,7,8. ^18, 19^

The Morisky Green test was used to assess adherence to pharmacological treatment. It consists of a series of 4 contrasting questions with a dichotomous yes/no response, which reflect the patient’s behaviour regarding compliance. The aim is to assess whether the patient adopts correct attitudes in relation to the treatment for their illness; it is assumed that if the attitudes are incorrect, the patient is non-compliant. The patient is compliant if he/she answers correctly to the 4 questions, i.e. No/Yes/No/No. ^20, 21^

The Valadez-Figueroa questionnaire was used to assess family support for patients with T2D, the interpretation of which is: low family support: 51-119 points, medium support: 120-187 and high support: 188-265 points. ^22^

For sampling and analysis of fasting glucose and glycosylated haemoglobin, the services of an external clinical laboratory were contracted. The results were delivered personally to each participant. Fasting glucose (mg/dL) and glycated haemoglobin (HbA1c %) were considered clinical outcome variables, as they allow the impact of the educational intervention on the metabolic control of patients with T2D to be assessed. Both variables were measured using standardized laboratory analysis. Fasting glucose was assessed in the pre- and post-intervention periods, while HbA1c was assessed as a complementary indicator of glycaemic control after the intervention.

Educational workshops were carried out with the participation of the patients included in the study, with a total of 103 participants and some family members. For the workshops, the population was distributed into two groups. Each group attended four sessions to develop the contents of knowledge of diabetes mellitus, its manifestations, complications and treatment, as well as physical activity and nutrition. It was considered achieved if the patient had a minimum of 3 sessions and in the post-tests showed a favorable change in the evaluated aspects. As there were difficulties for the workshops to be 100% face-to-face, workshops were supplemented by some virtual sessions via Zoom.

The unit of analysis was exclusively the patient with T2D. Family members participated solely as educational support. No data was collected or analyzed from family members.

The division was exclusively for logistical reasons (capacity and schedules), and both groups received the same intervention. There was no control group.

The variable ‘integral care’ is defined as the attendance of the patient with T2D at regular check-ups, with at least one medical examination per quarter, a nursing assessment and fasting blood glucose and glycosylated haemoglobin (HbA1c) results. Diabetes is considered uncontrolled if fasting blood glucose is >120 mg/dl and HbA1c is >7%. ^10, 11^ In this study, ‘integral care’ was not considered a single variable with a specific scale or score. It was conceptualized as a multidimensional construct that integrates clinical, behavioural, and psychosocial components of T2D management.

Operationally, integral care was assessed using specific and measurable indicators: level of knowledge (DKQ-24), eating habits, treatment adherence (Morisky-Green), family support (Valadez), fasting glucose, HbA1c, and attendance at medical and nursing check-ups.

To motivate and maintain participation, participants were entered into a WhatsApp group through which they were sent reminders to attend the workshops, the laboratory and feedback messages about what was discussed in addition to the workshop recordings.

The frequencies of the categorical variables were measured and the pre and post intervention results of each patient were evaluated. Likewise, for the quantitative variables, the Kolmogorov-Smirnov normality test was performed. ^23^ For data with normal distribution, the paired t-test was applied to evaluate the differences among the means of the scores obtained in the questionnaires on knowledge of DM, healthy diet, adherence to treatment and family support obtained before and after the educational intervention.

The Wilcoxon test was applied for data that did not have normal behavior. In addition, the analysis was complemented with the presentation of absolute and percentage frequencies to compare the changes in the before and after behavior in each category by applying the Mc Nemar test. A p-value < 0.05 was considered statistically significant, with a confidence level of 95%.

### Ethical considerations

The protocol entitled *‘Efficacy of an educational intervention in the integral care of patients with diabetes mellitus’* was submitted for consideration, comment, guidance and approval to the Research Ethics Committee prior to commencement of the research. This committee named *Research and Ethics Committee* of Health Care Network La Libertad - EsSALUD, approved the research project through Certificate N° 60 of 22 May 2023.

The research was conducted by professionals with appropriate scientific and ethical education, training and qualifications, with supervision by a competent and qualified medical practitioner.

The ethical principles of Helsinki were complied with data protection of the personal identity, privacy and confidentiality of the participants was complied with. ^24, 25^ This document is shown in extended data.

Participants signed a written and informed consent form, after explanation of the purpose of the research, the possible risks and benefits as well as the possibility to withdraw at any time, if deemed appropriate and without reprisal. ^24, 25^

## Results

Table 1 shows that females predominated (60.2%) and the mean age was 63.22 ± 7.96 years, with a higher proportion of adults aged 60 years or older (68.0%). 42.7% had higher education, and 70.9% were married or living with a partner. Regarding occupation, 44.7% were employed, and 35.9% were homemakers. 38.8% had had the disease for less than 5 years, while 35.9% had a disease duration of ≥10 years. Metformin was the most common treatment (72.8%), followed by its combination with glibenclamide (16.5%). The most prevalent comorbidity was hypertension (58.3%), followed by dyslipidemia (11.7%). 17.5% had no comorbidities.

**Table 1.** Characterization of patients with diabetes mellitus. Hospital I Albrecht. Essalud 2024.

Characteristics		n	%
Sex	Male	41	39.8%
	Female	62	60.2%
Age	(Average ± S)	(63.22 ± 7.96)	
	20-40 years	1	1.0%
	41-59 years	32	31.1%
	60-older	70	68.0%
Level of education	Illiterate	1	1.0%
	Primary School	17	16.5%
	Secondary School	29	28.2%
	Technician	11	10.7%
	Higher Education	44	42.7%
Marital Status	Single	9	8.7%
	Married/Cohabiting	73	70.9%
	Separated/Divorced	10	9.7%
	Widow (er)	11	10.7%
Occupation	Self-employed/employee	46	44.7%
	Housekeeper	37	35.9%
	Unemployed/retired	20	19.4%
Duration of illness	<5 years	40	38.8%
	5 to 9 years	26	25.2%
	10 to 15 years	19	18.4%
	>15 years	18	17.5%
Treatment	Metformin	75	72.8%
	Insulin	4	3.9%
	Metformin + insulin	3	2.9%
	Metformin + glibenclamide	17	16.5%
	glibenclamide	2	1.9%
	Others	2	1.9%
Comorbidity	None	18	17.5%
	Hypothyroidism	6	5.8%
	Dyslipidemia	12	11.7%
	Obesity	6	5.8%
	Anemia	3	2.9%
	Arterial hypertension	60	58.3%
	Chronic kidney disease	1	1.0%
	Others	25	24.3%

Table 2 shows a significant improvement in patients’ knowledge after the intervention. The mean knowledge improved with statistically significant difference (p < 0.001). With respect to family support there was a significant improvement in the level of support after the intervention. The difference between the means is statistically significant (p = 0.043), according to the Student’s t-test. The educational intervention not only increased average family support, but also homogenized the level of support among families, reducing the dispersion of scores. Regarding eating habits, it is evident that the educational intervention achieved a significant change as the percentage of patients with healthy eating habits increased from 3.9% to 12.6% (p = 0.022), indicating better adherence to a balanced diet (p = 0.022).

**Table 2.** Summary of effectiveness of the educational intervention in improving knowledge about diabetes mellitus, family support and healthy eating.

Indicator	n	Initial value Mean (DS)	After the intervention Mean (DS)	p
Knowledge about T2D	103	14.78 (2.65)	16.35 (2.21)	<0.001
Family support	103	171.1 (18.6)	171.5 (18.0)	0.043

Indicator	n	% (n)	% (n)	p
% Healthy eating habits	103	3.9% (4)	12.6% (13)	0.022

Table 3 shows that 51.5% of the participants stated that they did not comply with the prescribed treatment.

**Table 3.** Adherence to treatment in patients with T2D according to the Morisky Green test.

Adherence to treatment	N	%
Yes	50	48.5%
No	53	51.5%
Total	103	100.0%

In Table 4, McNemar’s test shows a significant difference (p < 0.01) in the proportion of patients complying with treatment before and after the intervention, suggesting that the intervention had a positive impact on improving treatment compliance. 76.8% of patients who were compliant with treatment maintained that behavior after the intervention. However, the proportion of patients who were initially noncompliant with treatment and failed to improve their compliance remained.

**Table 4.** Effectiveness of educational intervention in improving adherence to treatment in patients with T2D.

Compliance with the initial treatment	Compliance with treatment after the intervention				Total		Mc' Nemar test
	Yes		No				
	n	%	n	%	n	%	Sig.
Yes	43	76.8%	13	23.2%	56	54.4%	<0.01
No	0	0.0%	47	100.0%	47	45.6%	
**Total**	**43**	**41.7%**	**60**	**58.3%**	**103**	**100%**	

Table 5 shows that there was no improvement in glycemic control with the educational intervention. The mean fasting blood glucose went from 132.5 to 143.1 mg/dL according to the Wilcoxon test. The proportion of patients with adequate glycemic control dropped from 60.19% to 48.54% post intervention.

**Table 5.** Fasting blood glucose values and glycemic control in patients with T2D.

Indicator		Initial glycemia	Final glycemia	Wilcoxon	Sig.
Mean (mg/dL)		132.5	143.1	3774	<0.001
95% C.I. for the mean	Inferior	125.8	135.0		
	Superior	139.2	151.2		
Median		122.9	131.3		
Standard deviation		34.4	41.6		
Minimum		70	80		
Maximum		268.7	280.1		
Range		198.7	200.1		

Initial glycemic control	Glycemic control after the intervention				Total		Mc' Nemar test
	Yes		No				
	n	%	n	%	n	%	Sig.
Yes	46	74.19%	16	25.81%	62	60.19%	0.012
No	4	9.76%	37	90.24%	41	39.81%	
**Total**	**50**	**48.54%**	**53**	**51.46%**	**103**	**100.00%**	

In Table 6, we can see that 83.5% of patients have at least a quarterly follow-up with their physician and 44.7% with nursing, which indicates a regular frequency of follow-up according to EsSalud standards. Regarding HbA1c levels, the mean HbA1c is 7.54% for patients ≤ 69 years and 6.96% for patients ≥ 70 years. Only 33% of <70 years and 17.5% of >70, have good metabolic control.

**Table 6.** Integral management of patients with diabetes mellitus.

Integral control			n	%
Quarterly control with	Medical doctor	Yes	86	83.5%
		No	17	16.5%
	Nurse	Yes	46	44.7%
		No	57	55.3%
Reported adherence to treatment		Yes	50	48.5%
		No	53	51.5%
Glycosylated hemoglobin control by age group	Up to 69 years	(average ± S)	(7.54% ± 1.67%)	
		Good diabetes control	34	33.0%
		Risk of poor control	24	23.3%
		Poor control	24	23.3%
	70 years or older	(average ± S)	(6.96% ± 1.44%)	
		Good diabetes control	18	17.5%
		Risk of poor control	0	0.0%
		Poor control	3	2.9%

## Discussion

The population studied was predominantly female, with an average age of 63.22 years, higher education, married or cohabiting, and with a main occupation as an employee. Almost all patients had type 2 diabetes mellitus. As for the duration of the disease, the most frequent was less than 5 years. Two thirds of patients receive treatment with metformin, and the main comorbidity was arterial hypertension.

With respect to the characteristics of the population, in a Peruvian study on family support and glycemic control, the predominant population was female, with a mean age of 63.5 years ±12.10 years, occupation housewife followed by independent worker and the most common level of education was secondary education followed by primary education, ^24^ data very similar to those recorded in this study. Other studies also report a predominance of women, mean age of 53.14 years ±8.99, marital status married, ^8^ employed and with secondary education. ^6^ Other studies reported that the average age was 63 years. ^26, 27^ with a predominance of women (55.9%) but most patients had only primary education (58.9%), followed by those with secondary education (20.5%). ^26^

About gender, women appear to have a higher burden of risk factors at the time of diagnosis with T2D, particularly obesity, psychosocial stress, hormonal fluctuations, and bodily changes due to reproductive factors. Pregnancy can unmask preexisting metabolic abnormalities, leading to a diagnosis of gestational diabetes, which appears to be the most significant risk factor for progression to T2D in women. ^28^ On the other hand, aging leads to impaired beta-cell function and reduced adaptation to insulin resistance, increasing susceptibility to T2D. Additionally, older adults often experience comorbidities, frailty, and sarcopenia, which complicate diabetes management and increase prevalence. ^29^

Regarding the time of illness, 70.30% of patients had a time of illness less than or equal to 15 years (n = 111), and in 29.70%, it was more than 15 years. The average time of illness was 11 years, with a standard deviation of 9.51. ^26^ While in a study carried out in Colombia, the average time since the diagnosis of T2D was 11.3 ± 8.5 years. ^19^ Regarding this variable, it is important to mention that it is referred by the patient and that many of them are not clear about the time of illness, they tend to deny the disease or say that it is of recent onset.

In this study, we found a significant improvement in patients’ knowledge about T2D and its complications after the educational intervention (p < 0.001) and the level of adequate knowledge increased from 83.5% to 95% after the intervention (p < 0.001). About this variable, it has been described that those educational interventions are useful to change a certain behavior and to facilitate adequate self-care ^27^ and that structured educational programs for patients with T2D, especially aimed at older adults, have great potential; however, there is still room for improvement. ^30^ In the present study, the DKQ-24 17 was used to identify the knowledge that patients with T2D have of the disease, with the cut-off point of correctly answering ≥ 55% of the questions. The DKQ-24 is one of the most widely used questionnaires and the results indicate that more than 75% have adequate knowledge about their disease. Thus, a study conducted in ESSALUD with a population with similar characteristics in terms of sex, marital status and educational level, established a cut-off point of 75% for the results of the DKQ-24, and found that 17% of patients with T2D had adequate knowledge. ^31^

In this study, the educational intervention brought about a significant change in the dietary habits of patients with DM, with grater adherence to a healthy diet (p = 0.022). Some studies have found that people who are aware of the impact of diet tend to choose healthier food options to improve their glycemic control. ^32^ Evidence shows that group-based education on the impact of diet, physical activity, stress and sleep improves glucose regulation. ^33^ The Health Belief Theory explains that for patients to change behavior, they must perceive and understand the seriousness of the disease and the benefits of changing behavior ^34^ and adopting new habits. ^35^ They must also understand the effects of not changing. In this way, patient self-care is encouraged. ^34, 35^ From the health services, relevant and timely education, according to the Integrated Theory of Health Behavior Change, promotes knowledge and attitude change in patients as the only way to modify the behavior of the community.

When analyzing treatment compliance, 51.5% of the participants reported noncompliance. This finding is consistent with the 35-50% non-compliance rate reported in patients with chronic diseases, which is even higher in developing countries. ^36^ A Peruvian study reported that 74.3% of patients were not adherent to T2D treatment ^37^ Patients are considered to be adherent to T2D treatment when they have a compliance rate>=80% of pharmacotherapy and recommendations on diet, exercise and disease self-management, although in practice, less than 10% of patients with T2D meet these criteria. ^38^

In this regard, a panel of 58 experts pointed out that lack of adherence to treatment in patients with T2D leads to an increase in visits to emergency services, hospitalizations, failure to achieve therapeutic objectives, higher healthcare costs and a decrease in the patient’s quality of life. It also has negative consequences for the patient’s health and can mislead the treating physician as to the real effectiveness of the medication. ^38^

The educational intervention had a positive impact on improving family support, especially in families that initially had a high level of support. Research highlighted that family involvement is very important in the management of diabetes, as it resulted in a greater reduction in glycosylated hemoglobin HbA1c and a significant improvement in adherence to treatment. Intervention with family involvement is useful in diabetes management, especially when spouses or women are caregivers, ^39^ an effect that through multivariate analysis showed that family members who were spouses or women were strong predictors of better glycemic control. ^40^

The literature reviewed indicates that any educational intervention aimed at patients with T2D should involve family, friends and organizations in the community in which the patient lives, ideally within the framework of a government policy that provides the patient with a social support network. ^6^ It has also been described that the involvement of family members and caregivers is very important as one of the most influential factors in diabetes self-care practices and in the maintenance of long-term improvements. ^31^

This educational intervention had a positive impact on improving treatment adherence. Among patients who complied with their treatment, 76.8% maintained this behavior after the intervention, although the proportion of patients who initially did not comply with their treatment and failed to improve their compliance remained unchanged. This would require an evaluation of personal factors (age, employment status, marital status, and level of education) and cultural factors such as beliefs about the undesirable effects of medications used in the treatment of TD2 that could influence this outcome. ^35, 36^

A patient with T2D with good adherence to pharmacological treatment is more likely to also comply with adherence to the rest of the indications and change their eating, physical activity, and recreational habits. ^38^

Adherence to treatment has been shown to depend on several factors, including different individual and social levels, as most of them depend on the patient, the medication, the health care providers and the health care systems. Studies identified some of these factors for diabetes mellitus, such as depression, education level, gender, age, smoking status and employment status. Therefore, it is important to identify and pay attention to the effective factors involved in adherence to treatment in diabetic patients. ^36^ In this study, the factors influencing non-adherence to treatment were advanced age, single marital status, primary/secondary education level, and retirement from work.

The educational intervention showed no improvement in glycemia values and the proportion of patients with adequate glycemic control dropped from 60.19% to 48.54% after the intervention. It is likely that the time of the educational intervention should have been longer to evaluate this variable, since in other studies it takes between 8 to 12 months to achieve favorable changes in metabolic control ^8^ and maintaining them over time is more difficult. Therefore, follow-up periods of more than three years are recommended with periodic reevaluation of the patients at the end of the educational intervention. ^10^

Regarding integral control, most of patients comply with the EsSalud standard by performing at least a quarterly control with a physician and less than 45% comply with nursing control. Regarding HbA1c levels, the mean HbA1c is 7.54% for patients ≤ 69 years and 6.96% for patients ≥ 70 years. Only 33% of <70 years and 17.5% of >70, have good metabolic control. This is like that reported in a study in which patients with an average age of 63.9 years had HbA1c of 7.5%. ^27^

It has been described that it is important to adapt the general glycemic control goal (HbA1c < 7%) to each patient. Thus, in people without frailty and without risk of hypoglycemia, the HbA1c goal is < 6.5% and in the presence of frailty, comorbidities and risk of hypoglycemia, a less strict glycemic control goal may be appropriate (HbA1c 8-8.5%). ^41^ Other authors suggest that the HbA1c goal should also be adapted according to the patient’s age, years of disease and presence of complications. ^24^

It is important to consider that there are seven variables that are significantly associated with good diabetes mellitus control (P < 0.05): self-reported medication adherence, number of medications to which patients did not adhere, medication knowledge, diabetes knowledge, education level, total self-efficacy, and anxiety. ^41^

Scientific studies that have examined the topic of education in diabetes mellitus have systematically demonstrated that structured educational programs represent a fundamental pillar in disease management. The fact that a diabetic assumes a proactive role in the care of their condition not only favors its metabolic control but also increases patient adherence to their monitoring, contributing to improved nutritional status and clinical parameters related to their disease. ^6^ Traditionally, diabetes self-management education has involved talks or theoretical content; however, healthcare professionals generally feel they are not sufficiently equipped or have enough time to provide the necessary knowledge to ensure a healthy change in lifestyle; therefore, it would be important to use digital educational materials, reminders via text message or WhatsApp, mobile apps that combine different biometric and lifestyle parameters, and other recent innovations such as Diabetes Box to improve self-management and glycaemic control. ^42^ Furthermore, the experience of peer education using recreational workshop methodology has been reported, allowing patients to share their experiences regarding their knowledge and attitudes towards T2D in a more natural and effective way. ^43^

The studies reviewed highlight the need to rethink the educational approach used in Peruvian primary care centers for people with diabetes. There is a need to utilize mobile apps and telemonitoring, and to develop hybrid programmes (combining face-to-face and online elements) that promote healthy lifestyles focused on the patient as the primary agent of self-care, whilst also integrating family and community support.

Among the limitations of the study is that the use of structured questionnaires with closed questions could contain biased responses and that the patients cannot express everything they think. It was not possible to implement physical activity workshops because most patients had medical restrictions for physical activity. The duration of the intervention did not allow us to see the change in glycemia and HbA1c values. Also, the generalizability of the results may be limited because the subjects were selected from a single diabetes care program.

It is concluded that structured educational programmes are effective in improving the level of knowledge and lifestyles of patients with T2D, and that, therefore it is necessary to supplement/modernize these programmes by utilizing advances in tele-education, apps for people with diabetes, home monitoring, personalized lifestyle advice and the promotion of self-management to help patients change their health habits and improve glucose control.

## Authors’ contribution

Evelyn Goicochea-Ríos: Conceptualization, formal analysis, methodology, data collection, investigation, visualisation, writing – original draft preparation, writing – review & editing, funding acquisition.

Nélida Milly Otiniano: Methodology, data collection, formal analysis, writing – original draft preparation, writing.

## Data Availability

All data underlying the results are available as part of the article are available in the Zenodo repository, under the title “ DATA BASE Effectiveness of an educational intervention in the integral care of patients with diabetes mellitus” available from https://doi.org/10.5281/zenodo.14563599. ^44^ **Supplementary material** The supplementary material is available in the Zenodo repository, under the title. Effectiveness of an educational intervention in the integral care of patients with diabetes mellitus. ^44^ This project contains the following data: Database with results pre and post educational intervention, Diabetic family support assessment tool, Eating habits questionnaire, Educational Intervention workshops, Informed consent protocol, Morisky Green Test, Physical Activity Capability Questionnaire, Ethics committee certificate, 8-GATE-Knowledgequestionaire Spanish and format for collecting data. Data are available under the terms of the Creative Commons Attribution 4.0 International license (CC-BY 4.0).
