# Supplementary material for: Effectiveness of an Educational Intervention in the Integral Care of Patients with Diabetes Mellitus
Source: F1000Res. 2026 Oct 28;14:187. Originally published 2025 Feb 11. [Version 5] doi: 10.12688/f1000research.159626.5 (PMC13770169; doi:10.12688/f1000research.159626.5)
Supplement: Peer review history

# Reviewer report: Reviewer response for version 5

**DOI:** 10.5256/f1000research.213347.r535747

**Published:** 2026-11-14

**Authors:** Suyog Sindhu

**Affiliations:** 1 King George's Medical University, Lucknow, Uttar Pradesh, India

First of all, it's commendable on the part of authors to have thought of educational intervention in management of T2DM.

 However, I fail to understand as to why non-randomised sampling was chosen. Maybe this is the reason that mostly female gender was recruited!

 Authors are kindly requested to tell the status of formal Ethical permissions.

 As it is non-randomised design, possibility of bias creeps in, so I am not sure of the conclusions derived.

 Best Wishes to the authors to improve the manuscript.

Is the work clearly and accurately presented and does it cite the current literature?

Yes

If applicable, is the statistical analysis and its interpretation appropriate?

I cannot comment. A qualified statistician is required.

Are all the source data underlying the results available to ensure full reproducibility?

No

Is the study design appropriate and is the work technically sound?

No

Are the conclusions drawn adequately supported by the results?

Partly

Are sufficient details of methods and analysis provided to allow replication by others?

No

Reviewer Expertise:

Professor,Pharmacology

I confirm that I have read this submission and believe that I have an appropriate level of expertise to confirm that it is of an acceptable scientific standard, however I have significant reservations, as outlined above.

# Reviewer report: Reviewer response for version 4

**DOI:** 10.5256/f1000research.210366.r523277

**Published:** 2026-10-07

**Authors:** Saldy Yusuf

**Affiliations:** 1 Hasanuddin University, South Sulawesi, Indonesia

Dear Authors,

 Thank you for the fourth revision. We have a few substantive points that still need clarification and correction.

1. The Introduction now states the urgency of the study, but it still lacks an explicit research gap statement that contrasts what is already known with what remains unknown.
2. The study aim uses the term "efficacy", whereas the title uses "effectiveness". These are two different concepts, so please use one term consistently and make sure it fits the study design.
3. Table 1 includes a patient aged 20–40 years, which contradicts the inclusion criterion (adults aged 40–79 years). Please clarify.
4. In Table 4 (compliance with the initial treatment), no patient moved from non-compliant to compliant (No: 0, 0%), and the number of non-compliant patients after the intervention rose to 60 (58.3%). This is a negative change, yet the authors interpret it as a positive effect. Please clarify this interpretation.
5. Tables 3 and 4 are not consistent. The initial row of Table 4 records 56 patients (54.4%) as compliant with the initial treatment, whereas Table 3 records 50 patients (48.5%) as adherent to treatment. Are these the same measure, or do they differ in definition? Please clarify.
6. The number of sessions is inconsistent. The abstract states eight sessions, whereas the Methods state four sessions per group.
7. The abstract states "second quarter of 2024", whereas the Methods state "second semester".

Is the work clearly and accurately presented and does it cite the current literature?

Yes

If applicable, is the statistical analysis and its interpretation appropriate?

Partly

Are all the source data underlying the results available to ensure full reproducibility?

Partly

Is the study design appropriate and is the work technically sound?

Partly

Are the conclusions drawn adequately supported by the results?

No

Are sufficient details of methods and analysis provided to allow replication by others?

Partly

Reviewer Expertise:

Diabetes Mellittus, Diabetic Foot, Wound care

I confirm that I have read this submission and believe that I have an appropriate level of expertise to confirm that it is of an acceptable scientific standard, however I have significant reservations, as outlined above.

# Response

**Published:** 2026-10-21

**Authors:** Evelyn Goicochea Rios

**Affiliations:** Universidad Cesar Vallejo, Trujillo, La Libertad, Peru

Dear Reviewer:

 We sincerely appreciate your thorough evaluation of our article. We have carefully considered each comment and set out our responses below:

 1. The Introduction now states the urgency of the study, but it still lacks an explicit research gap statement that contrasts what is already known with what remains unknown.

 We reviewed the Introduction to distinguish more explicitly between the existing evidence on diabetes education and the areas requiring further evaluation in primary care. The revised text clarifies the contribution of our study, which assesses changes in diabetes-related knowledge, dietary habits, family support, treatment adherence and clinical indicators following an educational intervention amongst patients with type 2 diabetes mellitus.

 A paragraph has been added at the end of the Introduction to highlight this observation.

 2. The study aim uses the term "efficacy", whereas the title uses "effectiveness". These are two different concepts, so please use one term consistently and make sure it fits the study design.

 We agree with this observation, and have therefore standardized the terminology throughout the manuscript, using the term ‘effectiveness’ in a manner consistent with the title, the objective and the evaluation of the intervention in a primary care context.

 3. Table 1 includes a patient aged 20–40 years, which contradicts the inclusion criterion (adults aged 40–79 years). Please clarify.

 The database contained only one patient aged 40, so in response to your comment, we have removed the 20–40 age range and amended the following age range (40–59) to include that patient.

 4. In Table 4 (compliance with the initial treatment), no patient moved from non-compliant to compliant (No: 0, 0%), and the number of non-compliant patients after the intervention rose to 60 (58.3%). This is a negative change, yet the authors interpret it as a positive effect. Please clarify this interpretation.

 We thank the reviewer for identifying this inconsistency in the interpretation of Table 4. We recognize that our original interpretation incorrectly described the statistically significant difference detected by the McNemar test as evidence of a positive impact of the intervention on treatment adherence. Upon reviewing the results, we acknowledge that treatment adherence decreased from 54.4 % before the intervention to 41.7 % afterwards, whilst non-adherence increased from 45.6 % to 58.3 %.

 We have also amended the wording of this finding in the abstract and in the interpretation of Table 4. In the discussion section, we have removed the statement suggesting a positive impact of the intervention on treatment adherence (Discussion – paragraph 11).

 5. Tables 3 and 4 are not consistent. The initial row of Table 4 records 56 patients (54.4%) as compliant with the initial treatment, whereas Table 3 records 50 patients (48.5%) as adherent to treatment. Are these the same measure, or do they differ in definition? Please clarify.

 Table 3 sets out the responses given by each participant to the Morisky-Green test, whilst Table 4 sets out the data on treatment adherence before and after the educational intervention.

  Upon reviewing the database, we have verified that the correct data are 43 people who are adhering to treatment and 60 who are not; therefore, in response to your observation, we have amended these data in Table 3.

 6. The number of sessions is inconsistent. The abstract states eight sessions, whereas the Methods state four sessions per group.

 The audience capacity of the auditorium used for the educational sessions meant that participants had to be divided into two consecutive groups. Consequently, the full programme of workshops was completed over two months, but each participant attended four educational sessions. We have amended this information in the abstract.

 7. The abstract states "second quarter of 2024", whereas the Methods state "second semester".

 The research was carried out during the second semester of 2024. This information has been corrected in the abstract.

# Reviewer report: Reviewer response for version 3

**DOI:** 10.5256/f1000research.197420.r472084

**Published:** 2026-04-16

**Authors:** MARIA SOFIA CUBA-FUENTES

**Affiliations:** 1 Universidad Peruana Cayetano Heredia, San Martín de Porres, Peru

Based on the most recent version of the manuscript, I recommend acceptance with no further major revisions.

 The authors have adequately addressed the main concerns raised in previous rounds of review. In particular, they have: (1) clarified the definition of “integral care” and its relationship to the educational intervention; (2) provided a clearer description of the study population, inclusion and exclusion criteria, and sampling procedures; (3) specified the nature of the educational intervention and the variables assessed; and (4) added a participant flowchart that improves transparency and reproducibility of the methods. The introduction more clearly states the research aim, the methods section now contains sufficient detail for replication, and the discussion better situates the findings within the existing literature and reflects more explicitly on implications and limitations.

 Overall, the study is methodologically sound for its design, the results are presented in a coherent way, and the conclusions are supported by the data. The topic is relevant for diabetes care and health education in similar settings, and the manuscript now meets an acceptable scientific standard. I therefore support indexing of this version.

Is the work clearly and accurately presented and does it cite the current literature?

Partly

If applicable, is the statistical analysis and its interpretation appropriate?

Not applicable

Are all the source data underlying the results available to ensure full reproducibility?

Partly

Is the study design appropriate and is the work technically sound?

Partly

Are the conclusions drawn adequately supported by the results?

Yes

Are sufficient details of methods and analysis provided to allow replication by others?

Partly

Reviewer Expertise:

Family Medicine, Primary Care

I confirm that I have read this submission and believe that I have an appropriate level of expertise to confirm that it is of an acceptable scientific standard.

# Reviewer report: Reviewer response for version 2

**DOI:** 10.5256/f1000research.196200.r458047

**Published:** 2026-03-09

**Authors:** MARIA SOFIA CUBA-FUENTES

**Affiliations:** 1 Universidad Peruana Cayetano Heredia, San Martín de Porres, Peru

The manuscript addresses an important topic and presents relevant findings for the field. However, the research objective could be stated more explicitly in the introduction. While the manuscript provides contextual background, the specific research question and its contribution to the existing literature should be more clearly articulated. Clarifying the conceptual framework and linking it more explicitly to current research would strengthen the overall narrative.

 Additional detail is needed to ensure transparency and reproducibility. In particular, the manuscript would benefit from a clearer description of:

- how participants or data sources were selected
- inclusion and exclusion criteria
- the procedures used for data collection
- the analytical framework applied.

Providing this information would allow readers to better understand the methodological rigor of the study and facilitate replication.

 The results presented in the manuscript are informative; however, the interpretation of these findings could be expanded. In particular, the discussion would benefit from deeper engagement with recent literature addressing similar topics. Placing the results within the broader international context would help highlight the contribution of the study and clarify how it advances current knowledge.

 The discussion section could be further developed to explore the implications of the findings for policy, practice, or future research. 

 Although some limitations are acknowledged, this section could be expanded. It would be helpful for the authors to discuss potential sources of bias, limitations related to data availability or methodological constraints, and how these factors may affect the interpretation of the results.

 This manuscript addresses a relevant topic and provides useful insights for the field. With improvements in methodological transparency, clearer articulation of the research objectives, and a more developed discussion of the implications of the findings, the manuscript would make a valuable contribution to the literature.

Is the work clearly and accurately presented and does it cite the current literature?

Partly

If applicable, is the statistical analysis and its interpretation appropriate?

Not applicable

Are all the source data underlying the results available to ensure full reproducibility?

Partly

Is the study design appropriate and is the work technically sound?

Partly

Are the conclusions drawn adequately supported by the results?

Yes

Are sufficient details of methods and analysis provided to allow replication by others?

Partly

Reviewer Expertise:

Family Medicine, Primary Care

I confirm that I have read this submission and believe that I have an appropriate level of expertise to confirm that it is of an acceptable scientific standard, however I have significant reservations, as outlined above.

# Response

**Published:** 2026-03-20

**Authors:** Evelyn Goicochea Rios

**Affiliations:** Universidad Cesar Vallejo, Trujillo, La Libertad, Peru

Dear Dr Sofia Cuba,

 We have received your comments and recommendations, and we would like to respond to them as follows:

1. The manuscript addresses an important topic and presents relevant findings for the field. However, the research objective could be stated more explicitly in the introduction. While the manuscript provides contextual background, the specific research question and its contribution to the existing literature should be more clearly articulated. Clarifying the conceptual framework and linking it more explicitly to current research would strengthen the overall narrative.

- The wording of the introduction has been improved and the aim of the research has been explicitly stated.

 

- 2.Additional detail is needed to ensure transparency and reproducibility. In particular, the manuscript would benefit from a clearer description of: how participants or data sources were selected, inclusion and exclusion criteria, the procedures used for data collection, the analytical framework applied.

 

- This information can be found in the methodology section, and we have included Figure 1, which details information on the participants, the inclusion and exclusion criteria, and the population on which the study was conducted.Information on the procedure used for data collection is provided in the ‘Techniques and Procedures’ section and the data collection form has been outlined.

3. Providing this information would allow readers to better understand the methodological rigor of the study and facilitate replication.

 The results presented in the manuscript are informative; however, the interpretation of these findings could be expanded. In particular, the discussion would benefit from deeper engagement with recent literature addressing similar topics. Placing the results within the broader international context would help highlight the contribution of the study and clarify how it advances current knowledge.

- The results are discussed and compared with the findings of other studies in the discussion chapter, and the literature on education for patients with diabetes has been expanded.

4. The discussion section could be further developed to explore .the implications of the findings for policy, practice, or future research. 

- This suggestion was taken on board

5. Although some limitations are acknowledged, this section could be expanded. It would be helpful for the authors to discuss potential sources of bias, limitations related to data availability or methodological constraints, and how these factors may affect the interpretation of the results.

- The report also mentions the potential biases arising from questionnaires containing closed-ended questions and has included the limitations regarding the capacity of the institutional auditorium

We look forward to your comments

# Reviewer report: Reviewer response for version 2

**DOI:** 10.5256/f1000research.196200.r458053

**Published:** 2026-02-16

**Authors:** Saldy Yusuf

**Affiliations:** 1 Hasanuddin University, South Sulawesi, Indonesia

Interesting manuscript, but I note some points that need revision.

 Background:

 since the central topic is integral care of patients with DM, how did author define this term, and integrate educational intervention.

 The background will be clear if authors explain what is unknown regarding integral care which make current study different with similar previous studies.

 We also found no clear aim statement at the end of background.

 Method

 We suggest authors to present participants flow chart (from recruitment to analysis, including its drop out reason).

 Educational workshop:

 What is the participants of current study? patient, family, or both? why there is some family members included? its inconsistent with previous information.

 For the workshops, the population was distributed in two groups. Its unclear why authors allocated participants in two groups, while in method current study reported as pre and post intervention (without control), all of tables also indicate no control group (only single group).

 In unclear in method, delivery of educational workshop, does it face to face, zoom, or hybrid (combine) approach.

 For the variable integral care, its unclear for readership, whether integral care as research variable or as intervention modality?

 authors has used some instruments:

 1. Diabetes Knowledge Questionnaire - DKQ 2417.

 2. The eating style and physical activity questionnaire

 3. The Morisky-Green test

 4. Valadez test

 Do these instruments use  independent variables?

 and also evaluate fasting glucose and glycosylated haemoglobin (does it research outcome?), please clarify.

 Then, how about the variable integral care (does it independent of dependent variable?) how did author measure, what is the scale or score?

 Please explain unit analysis, type of data, the reason for selecting statistical test.

 Table 5 unclear

 Fasting glucose or glycosylated haemoglobin?

 We found no conclusion statement.

Is the work clearly and accurately presented and does it cite the current literature?

Yes

If applicable, is the statistical analysis and its interpretation appropriate?

Partly

Are all the source data underlying the results available to ensure full reproducibility?

Partly

Is the study design appropriate and is the work technically sound?

Partly

Are the conclusions drawn adequately supported by the results?

No

Are sufficient details of methods and analysis provided to allow replication by others?

Partly

Reviewer Expertise:

diabetes mellitus, diabetic foot, foot care, wound care, wound management, clinical nursing

I confirm that I have read this submission and believe that I have an appropriate level of expertise to confirm that it is of an acceptable scientific standard, however I have significant reservations, as outlined above.

# Response

**Published:** 2026-03-03

**Authors:** NELIDA MILLY OTINIANO

Dear reviewer, we appreciate your comments and suggestions and respond below.

 1. Background:

 Since the central topic is integral care of patients with DM, how did author define this term, and integrate educational intervention. The background will be clear if authors explain what is unknown regarding integral care which make current study different with similar previous studies. We also found no clear aim statement at the end of background.

**Response**: The definition of the variable ‘integral care’ was considered if the patient underwent periodic check-ups, with at least one medical examination per quarter, and underwent fasting blood glucose and HbA1c tests. Abnormal values were considered to be >120 mg/dl and >7% respectively.

 - The definition of the variable ‘integral care’ can be found in Procedures and techniques.

 - The educational intervention was considered part of the protocol for caring for patients with diabetes mellitus. It is described in the background section.

 - The general objective was to evaluate the efficacy of an educational intervention in the integral care of the patient with diabetes mellitus through the pre and post intervention analysis of knowledge about DM, dietary habits, type and frequency of physical activity, metabolic control, adherence to treatment and family support”… (in the last paragraph of the background section)

 2. Method: 

 We suggest authors to present participants flow chart (from recruitment to analysis, including its drop out reason).

**Response**: The flowchart was placed in the manuscript.

 3. Educational workshop:

 What is the participants of current study? patient, family, or both? why there is some family members included? its inconsistent with previous information. For the workshops, the population was distributed in two groups. Its unclear why authors allocated participants in two groups, while in method current study reported as pre and post intervention (without control), all of tables also indicate no control group (only single group). In unclear in method, delivery of educational workshop, does it face to face, zoom, or hybrid (combine) approach.

**Response**: The unit of analysis was exclusively the patient with DM2. Family members participated solely as educational support. No data was collected or analyzed from family members.

 - The division was exclusively for logistical reasons (capacity and schedules), and both groups received the same intervention. There was no control group.

 - The article mentions that the workshops were predominantly in-person, supplemented by some virtual sessions via Zoom.

 - integral care is one of the variables under investigation and is also a component of the person-centred care model for people with diabetes mellitus in Peru.

 4. Authors has used some instruments:

 - Diabetes Knowledge Questionnaire - DKQ 2417.

 - The eating style and physical activity questionnaire

 - The Morisky-Green test

 - Valadez test

 Do these instruments use  independent variables?

**Response:**  Instruments used measure dependent variables: 

 * Knowledge (DKQ-24)

 * Eating habits

 * Treatment adherence (Morisky-Green)

 * Family support (Valadez test)

 4.1 and also evaluate fasting glucose and glycosylated haemoglobin (does it research outcome?), please clarify.

**Response**: Fasting glucose (mg/dL) and glycated haemoglobin HbA1c (%) were considered clinical outcome variables, as they allow the impact of the educational intervention on the metabolic control of patients with type 2 diabetes mellitus to be assessed.

 - Both variables were measured using standardized laboratory analysis. Fasting glucose was assessed in the pre- and post-intervention periods, while HbA1c was assessed as a complementary indicator of glycaemic control after the intervention.

 4.2 Then, how about the variable integral care (does it independent of dependent variable?) how did author measure, what is the scale or score?

**Response:** In this study, ‘integral care’ was not considered a single variable with a specific scale or score. It was conceptualized as a multidimensional construct that integrates clinical, behavioural, and psychosocial components of type 2 diabetes mellitus management.

 - Operationally, comprehensive care was assessed using specific and measurable indicators: level of knowledge (DKQ-24), eating habits, treatment adherence (Morisky-Green), family support (Valadez), fasting glucose, HbA1c, and attendance at medical and nursing check-ups.

 Therefore, comprehensive care was analyzed through the joint behavior of these dependent variables.

 5. Please explain unit analysis, type of data, the reason for selecting statistical test.

 The variables analyzed were classified according to their nature:

**Response**: Continuous quantitative variables: fasting glucose (mg/dL), glycosylated haemoglobin HbA1c (%),         DKQ-24 questionnaire score and family support test score (Valadez).

 - Discrete quantitative variables: number of quarterly check-ups.

 - Dichotomous categorical variables: adherence to treatment (yes/no), eating habits (healthy/unhealthy), attendance at medical and nursing check-ups (yes/no).

 - The normality of the quantitative variables was assessed using the Kolmogorov-Smirnov test and graphical analysis.

 For the comparison of pre- and post-intervention means:

 - - The Student's t-test for related samples was used when the variable was normally distributed.

 - The non-parametric Wilcoxon signed-rank test was used when the distribution was not normal.

 - For the analysis of paired categorical variables (pre- and post-), the McNemar test was used, which is appropriate for comparing proportions in dependent samples.

 A statistical significance level of p < 0.05 was established, and 95% confidence intervals were reported.

 6. Table 5 unclear

**Response:** Table 5 shows only fasting blood glucose values (mg/dL) compared before and after theintervention.

 7. We found no conclusion statement.

**Response**: The conclusion appears at the end of the discussion:

  ‘It is concluded that structured educational programs are effective in improving the knowledge and lifestyles of patients with T2D and that, therefore, these programs need to be reinforced in order to improve the comprehensive care of these patients’...
